# SINEUP non-coding RNAs rescue defective frataxin expression and activity in a cellular model of Friedreich's Ataxia

**DOI:** 10.1093/nar/gkz798

**Published:** 2019-10-04

**Authors:** Carlotta Bon, Riccardo Luffarelli, Roberta Russo, Silvia Fortuni, Bianca Pierattini, Chiara Santulli, Cristina Fimiani, Francesca Persichetti, Diego Cotella, Antonello Mallamaci, Claudio Santoro, Piero Carninci, Stefano Espinoza, Roberto Testi, Silvia Zucchelli, Ivano Condò, Stefano Gustincich

**Affiliations:** 1 Central RNA Laboratory, Istituto Italiano di Tecnologia (IIT), Genova, Italy; 2 Area of Neuroscience, International School for Advanced Studies (SISSA), Italy; 3 Department of Biomedicine and Prevention, Laboratory of Signal Transduction, University of Rome Tor Vergata, Rome, Italy; 4 Department of Health Sciences and Interdisciplinary Research Center of Autoimmune Diseases (IRCAD), University of Piemonte Orientale (UPO), Novara, Italy; 5 RIKEN Center for Life Science Technologies, Division of Genomic Technologies, Yokohama, Kanagawa, Japan

## Abstract

Friedreich's ataxia (FRDA) is an untreatable disorder with neuro- and cardio-degenerative progression. This monogenic disease is caused by the hyper-expansion of naturally occurring GAA repeats in the first intron of the *FXN* gene, encoding for frataxin, a protein implicated in the biogenesis of iron-sulfur clusters. As the genetic defect interferes with *FXN* transcription, FRDA patients express a normal frataxin protein but at insufficient levels. Thus, current therapeutic strategies are mostly aimed to restore physiological *FXN* expression. We have previously described SINEUPs, natural and synthetic antisense long non-coding RNAs, which promote translation of partially overlapping mRNAs through the activity of an embedded SINEB2 domain. Here, by *in vitro* screening, we have identified a number of SINEUPs targeting human *FXN* mRNA and capable to up-regulate frataxin protein to physiological amounts acting at the post-transcriptional level. Furthermore, *FXN*-specific SINEUPs promote the recovery of disease-associated mitochondrial aconitase defects in FRDA-derived cells. In summary, we provide evidence that SINEUPs may be the first gene-specific therapeutic approach to activate *FXN* translation in FRDA and, more broadly, a novel scalable platform to develop new RNA-based therapies for haploinsufficient diseases.

## INTRODUCTION

Friedreich's ataxia (FRDA) is a life-threatening monogenic disease with neuro- and cardio-degenerative progression (1). It represents the most frequent type of inherited ataxia, affecting >15 000 patients in Europe and North America. Patients typically show degeneration of large sensory neurons of the dorsal root ganglia, of Betz pyramidal neurons of the cerebral cortex and lateral cortico-spinal and spinocerebellar tracts, as well as lesions in the dentate nucleus of the cerebellum (2). In addition, non-neurological degeneration causes hypertrophic cardiomyopathy and increased incidence of diabetes mellitus. Neurodegenerative motor symptoms typically appear before adolescence with progressive gait instability and loss of coordination, while the cardiac component of the disease causes premature mortality at a mean age of 40 years (3). Almost all FRDA patients carry an intronic homozygous expansion of natural GAA repeats located in the frataxin (*FXN*) gene (4). The human *FXN* locus contains normally from 10 to 66 GAA-triplet repeats within the first intron, whereas FRDA individuals have a hyper-expansion of such repeats, up to 1700 triplets. In a small percentage of cases, however, patients are compound heterozygotes for GAA expansion on one *FXN* allele and a second allele with a small insertion, deletion or point mutation in *FXN* open reading frame (5). Longer hyper-expansions result in a more severe phenotype with an earlier onset and faster progression (6,7). GAA repeat expansions impair *FXN* transcription by inducing the formation of triple helical DNA structures (sticky DNA) (8), persistent DNA/RNA hybrids (R-loops) (9) and specific epigenetic modifications (10,11). The *FXN* gene encodes for the precursor of frataxin, a small iron-binding protein, that is mainly, but not exclusively, confined inside the mitochondrial matrix (12–14), where it is converted into the functional mature form (15). Although its primary function is still debated (16), mature frataxin is a key component of the Iron-Sulfur Cluster (ISC) biosynthetic apparatus (17–20), which provides the essential cofactor to all ISC-dependent enzymes of the cell (21,22). As consequence of insufficient *FXN* expression, defective ISC biosynthesis triggers a series of vicious cycles leading to deregulated intracellular iron homeostasis, impaired mitochondrial electron transport chain and higher sensitivity to trigger oxidant- and stress-induced cell death (23–25).

Currently, there are no therapies to treat the disease or prevent its progression. The most promising approaches point to restore sufficient frataxin levels (26), mostly by enhancing *FXN* transcription. Among them, interferon gamma (IFN-γ) (27) and dyclonine (28) have been identified as encouraging candidates by drug repositioning programs. Synthetic histone deacetylase (HDAC) inhibitors have been described to increase *FXN* mRNA in FRDA-derived cells and in FRDA animal models (29,30). More recently, synthetic nucleic acids were successfully employed targeting GAA repeats, acting as DNA:RNA hybrids (R-loops) inhibitors (31). Moreover, polyamide-based transcription factors capable of binding GAA microsatellite were developed (32). Interestingly, protein replacement therapy based on Trans-Activator of Transcription (TAT) fusion frataxin (TAT-frataxin) delivery (33), and frataxin degradation prevention by a class of ubiquitin-competing small molecules (34), have been recently proposed as potential treatments targeting the frataxin polypeptide. Finally, an effective gene replacement strategy in the FRDA mouse model opened new opportunities for gene therapy (35). However, recent data has proved that prolonged over-expression at non-physiological levels of frataxin affects cellular metabolism, leading to a significant increase of oxidative stress and labile iron pool levels (36). These cellular alterations are similar to those observed when the gene is partly silenced, as occurs in FRDA patients. These results suggest that any long-term therapeutic intervention must finely tune frataxin protein levels within a physiological range.

RNA therapeutics is of great interest for its potentials in enlarging the range of druggable targets. The ability to manipulate gene expression for every transcript could revolutionize molecular medicine with the tremendous advantage of scalability. A large group of diseases, including FRDA, would strongly benefit from the discovery of RNAs able to increase gene expression and restore physiological transcription and/or translation of a specific target when low expression is pathogenic.

**Figure 1.**
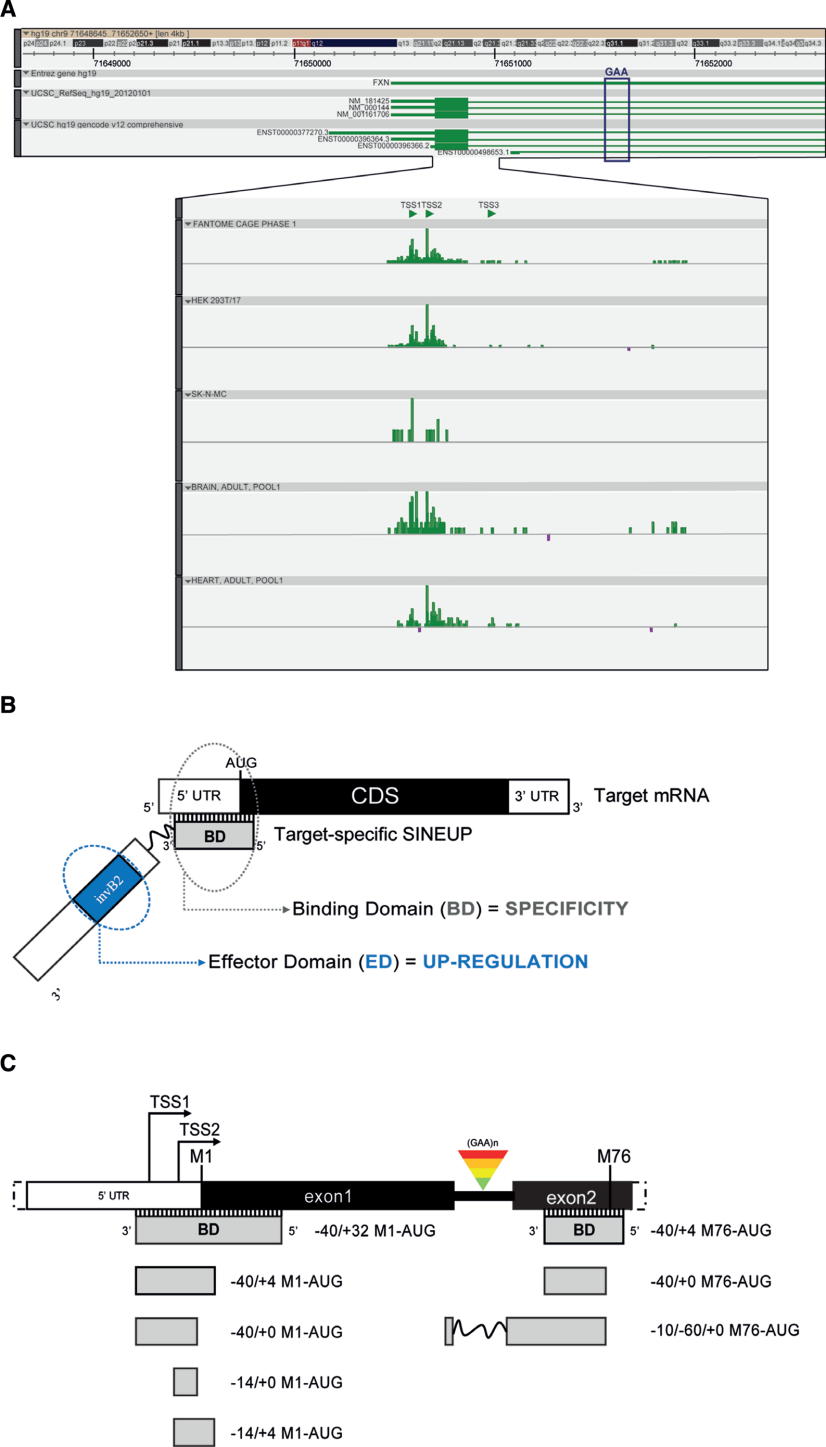
**Design of synthetic SINEUPs to target *FXN* mRNA**. (**A**) ZENBU genome browser view of *FXN* locus, showing *FXN* alternative TSS usage (TSS1, TSS2 and TSS3) in FANTOM5 samples and selected libraries. *FXN* reference sequences and Gencode annotated transcripts are indicated. The genomic position of the GAA triplet repeat region is highlighted (black box). (**B**) Schematic representation of SINEUPs functional domains. The binding domain (BD, gray) provides SINEUP specificity and it is in antisense orientation to the sense protein-coding mRNA (Target mRNA). The inverted SINEB2 element (invB2) is the effector domain (ED, light blue) and confers enhancement of protein synthesis. 5′ to 3′ orientation of sense and antisense RNA molecules is indicated. Structural elements of target mRNA are shown: 5′ untranslated region (5′UTR, white), coding sequence (CDS, black) and 3′ untranslated region (3′UTR, white). The scheme is not drawn in scale. (**C**) Scheme of human *FXN* gene (5′-end, white) and BDs (grey) design of synthetic *SINEUP-FXN* targeting the initiating M1-AUG and the M76-AUG downstream GAA expansions. The numbering refers to the position according to the methionine (i.e. −40/+32, from 40 nucleotides upstream and to 32 nucleotides downstream the M1-AUG). The scheme is not drawn in scale.

AS Uchl1 was previously isolated as a long non-coding RNA (lncRNA) antisense to Ubiquitin carboxy-terminal hydrolase L1 (*Uchl1/Park5*), a protein-coding mouse gene. This sense/antisense pair presents a 5′ head-to-head configuration with AS Uchl1 overlapping the sense gene for 72 nucleotides (nts) around the AUG translation initiation codon. The overlapping region starts from +32 nts ending −40 nts to AUG (A is +1) (indicated as −40/+32). The non-overlapping part of AS Uchl1 contains embedded Transposable Elements including a SINEB2 (short interspersed nuclear element of B2 subfamily) sequence in inverted orientation. Interestingly, overexpression of full length AS Uchl1 RNA increases UCHL1 protein amounts acting at the post-transcriptional level (37). This activity has been proved to reside in the antisense region and in the embedded SINEB2 element. Since this ability is shared with several mouse antisense lncRNAs, AS Uchl1 has been considered the representative member of a new functional class of natural and synthetic lncRNAs, named SINEUPs, since through the activity of an embedded inverted SINEB2 (invSINEB2) sequence they UP-regulate translation. Synthetic SINEUPs require two functional domains for their translational enhancement activity. In the non-overlapping portion, the invSINEB2 sequence acts as Effector Domain (ED). In the 5′, the overlapping region represents the Binding Domain (BD) and provides target selection and specificity (Figure 1B). Given knowledge of the exact Transcription Start Site (TSS) and consequently of the 5′ untranslated region (UTRs) of target mRNA, synthetic SINEUPs can be designed virtually for any gene of interest to increase target protein levels.

While the natural anatomy of the antisense sequence found in AS Uchl1 has been considered a model for BD design in synthetic SINEUPs, additional BD domains have been successfully tested. Furthermore, the exclusive combination of ED and BD sequences, called miniSINEUPs, promotes target protein up-regulation at post-transcriptional level with equal efficiency proving the other regions of AS Uchl1 dispensable for translation enhancing activity. Synthetic SINEUPs and miniSINEUPs have been shown to be effective *in vitro* on a wide spectrum of targets and in different cell types (38–40). Most importantly, synthetic SINEUPs have been proved to correct *in vivo* defective gene expression with amelioration of eye and brain phenotypes in a fish model of human Microphthalmia with Linear Skin defects syndrome, a disease caused by haploinsufficient dosage of the mitochondrial protein COX7b (41).

Here, we show that *SINEUP-FXN* and *miniSINEUP-FXN* enhance endogenous quantities of mature frataxin in a variety of human cell lines. SINEUP-mediated up-regulation is even more pronounced in cells derived from FRDA patients, where GAA expansion causes defective expression and functional insufficiency. A 2-fold increase in frataxin quantities is measured both in fibroblasts and in lymphoblasts, as induced by four independent SINEUPs targeting mRNA regions located pre- and post-GAA expansion. Most importantly, in FRDA cells, SINEUPs functionally rescue defects in mitochondrial aconitase activity, one of the hallmarks of the disease. Altogether, these results pave the way for the use of SINEUPs in RNA therapeutics of FRDA as well as a scalable platform for haploinsufficiencies.

## MATERIALS AND METHODS

### Oligonucleotides

The complete list of oligonucleotides used for cloning, quantitative real-time PCR experiments and lentivirus titration is included in Supplementary material (Supplementary Table S1).

### Constructs

*SINEUP-FXN* were generated using pcDNA 3.1(−)- Δ5′-AS Uchl1 as backbone (37) lacking the region of overlap (BD) to Uchl1 and retaining AS Uchl1 ED. BDs were designed in antisense orientation to the most widely expressed human *FXN* mRNA, targeting the first or the second AUG, with longer or shorter overlapping region (37). Oligonucleotides were annealed and cloned into recipient plasmid. For the plasmid-driven expression of *miniSINEUP-FXN*, we constructed a DNA cassette containing the H1 RNA polymerase III promoter followed by the BDs of *SINEUP-FXN* of interest, the invSINEB2 of AS Uchl1 (38), and a minimal polyadenylation signal (42). The cassette was cloned into the AseI restriction site of pEGFP-C2 vector (Clontech), with the H1 promoter oriented in the opposite direction with respect to the CMV promoter. The resulting family of plasmids is designed to constitutively express a miniSINEUP of interest and the enhanced Green Fluorescent Protein (EGFP). All SINEUP and miniSINEUP-containing vectors were verified by sequencing.

### Cell lines

HEK 293T/17 were obtained from ATCC^®^ (Cat. No. CRL-11268™) and maintained in culture with Dulbecco's modified Eagle's medium (DMEM) GlutaMAX™ Supplement (Gibco by Life Technologies, Cat. No. 41090-028) supplemented with 10% fetal bovine serum (Euroclone, Cat. No. ECS0180L) and 1% antibiotics (penicillin/streptomycin), as suggested by the vendor. SH-SY5Y cells were obtained from ATCC^®^ (Cat. No. CRL-2266™) and maintained in culture with RPMI supplemented with GlutaMAX, 10% fetal bovine serum not inactivated (Euroclone, Cat. No. ECS0180L) and 1% antibiotics (penicillin/streptomycin). Human GM04078 fibroblasts, from a clinically affected FRDA patient homozygous for the GAA expansion in the *FXN* gene with alleles of approximately 541 and 420 repeats, were obtained from NIGMS Human Genetic Repository, Coriell Institute for Medical Research (Camden, NJ, USA). Cells were maintained in culture with minimum essential medium (MEM) HEPES, GlutaMAX™ Supplement (Gibco by Life Technologies, Cat. No. 42360024) supplemented with 15% fetal bovine serum heat inactivated (Euroclone, Cat. No. ECS0180L), 1% non-essential amino acids and 1% antibiotics (penicillin/streptomycin). Human GM16214 lymphoblasts from a clinically affected FRDA patient homozygous for the GAA expansion in the *FXN* gene with alleles containing approximately 700 and 600 repeats and human GM16215 lymphoblasts from a clinically unaffected parent with only one allele containing 830 repeats (mother of affected GM16214), were obtained from NIGMS Human Genetic Repository, Coriell Institute for Medical Research (Camden, NJ, USA). Both cell lines were maintained in culture with RPMI 1640 Medium (Euroclone ECB9006) supplemented with 15% fetal bovine serum heat inactivated (Hyclone CHA1115L),100 U/mg penicillin/streptomycin (Euroclone ECB3001D) and 2 mM l-glutamine (Euroclone ECB3000D).

### Transfections

HEK 293T/17 and SH-SY5Y cells were plated in six-well plates the day before transfection at 60% confluency (4 × 10^5^ cells/well) and transfected with 4 μg of SINEUPs or miniSINEUPs encoding plasmids using Lipofectamine^®^ 2000 (Invitrogen™ by Life Technologies, Cat. No. 11668019) and following manufacturer's instructions. Cells were collected 48 hours after transfection. RNA and protein were obtained from the same transfection in each replica.

### Recombinant lentivirus production and titration

Selected *miniSINEUP-FXN* were cloned into a TetON-controlled lentiviral vector, based on the pCCLsin.PPT.hPGK.EGFP Wpre backbone (43). Recombinant third generation self-inactivating (SIN) lentiviruses were produced and titrated as previously described (43). Briefly, HEK 293T/17 cells were transfected using LipoD293™ DNA In Vitro Transfection Reagent (SignaGen Laboratories, Cat. No. SL100668-5) with the transfer vector plasmid plus three auxiliary plasmids (pMD2 VSV.G; pMDLg/pRRE; pRSV-REV). The conditioned medium was collected after 24 and 48 h, filtered and ultra-centrifuged at 50 000 RCF on a fixed angle rotor (JA 25.50 Beckmann Coulter) for 165 min at 4°C. Viral pellets were resuspended in DPBS without BSA (Gibco, Cat. No. 14190250). miniSINEUP-expressing lentiviral particles were titrated by real time quantitative PCR after infection of HEK293T/17 cells. One end-point fluorescence-titrated lentivirus was included in each PCR titration session and PCR-titers were converted into fluorescence-equivalent titers throughout the study.

### Infection of Human FRDA fibroblasts

At least 8.5 × 10^5^ FRDA fibroblasts were plated onto a 100 mm plate with medium supplemented with Hexadimethrine bromide at a final concentration of 0.009 μg/ml and infected with the appropriate miniSINEUP-expressing lentiviral vector (multiplicity of infection, MOI 10) together with trans-activating lentiviral vector (MOI 10). Cells were treated with doxycycline (1 μg/ml) every 48 h after transduction and collected after 4 days of treatment. RNA and proteins were obtained from the same infection in each replica.

### Stable transfections of FRDA lymphoblasts

FRDA GM16214 lymphoblasts were transfected by electroporation as already described (13). Briefly, 15 × 10^6^ cells were incubated in 0.4 ml of RPMI 1640 for 10 min on ice with 30 μg of *miniSINEUP-FXN* constructs and the empty vector (ctrl). After electroporation at 260 V/950 microfarads (Bio-Rad GenePulser II), cells were left 30 min on ice and resuspended in 5 ml of complete RPMI 1640 medium. After 4 h, live cells were recovered by Lympholyte-Human (Cedarlane Laboratories) density gradient centrifugation and re-plated. Stable transfectants were obtained from cultures in selection medium containing 600 μg /ml G418 (Sigma) for at least 15 days.

### Aconitase assays

Whole-cell extracts from GM16215 lymphoblasts, GM16214 lymphoblasts and GM16214 lymphoblasts stably expressing miniSINEUPs were prepared in ice-cold CelLytic M buffer (Sigma-Aldrich) supplemented with 2 mM sodium citrate and Complete EDTA-free protease inhibitor cocktail (Roche Diagnostic). Spectrophotometric aconitase assays were performed at 25°C with 100 μg of cell extracts using the BIOXYTECH Aconitase-340 Assay (OxisResearch 21041). Spectrophotometric citrate synthase activities were assessed at 25°C with 10 μg of cell extracts using the Citrate Synthase Assay Kit (Sigma-Aldrich CS0720). For the calculation of the activities, one unit of enzyme was expressed as the amount of protein that converted 1 μmol of substrate per minute at 25°C.

### Off-targets prediction

Original sequences of *FXN* mRNA were aligned by using Basic Local Alignment Search Tool (BLASTN) of Ensembl genome browser against human cDNA database (Ensemble's annotation repository based on GRCh38 assembly). The alignment sensitivity and scoring system were modified based on short sequence parameters, filtered for sequence similarity and matching consistency with the hit. Results were further filtered to discard non-functional genes. Selected off-targets are listed in Figure 5A.

### Western blot

Transfected HEK 293T/17 and SH-SY5Y cells were washed twice and collected in PBS 1X. After 5 min centrifugation at 500 RCF, cell pellets were directly lysed in 300 μl of Laemmli sample buffer, briefly sonicated, boiled and loaded 10–20 μl/each sample on 10–15% SDS-PAGE gel. Infected human FRDA fibroblasts were collected in PBS 1×. After 5-min centrifugation at 500 RCF, cell pellets were dissolved in 100 μl of Laemmli sample buffer, briefly sonicated, boiled and loaded 20 μl/each sample on 10–15% SDS-PAGE gel. Proteins were transferred to nitrocellulose membrane (Amersham™, Cat. No. GEH10600001) for 1 h at 100 V. Membranes were blocked with 3% Bovine Serum Albumin (SIGMA, Cat. No. A2058) in TBST 1× solution for 1 h at room temperature and then incubated with primary antibodies. The following antibodies were used: anti-FXN 4 μg/ml (Abcam, Cat. No. 18A5DB1) overnight at 4°C followed by 1-h incubation at room temperature with horseradish peroxidase-conjugated goat anti-mouse antibody (DakoCytomation, Glostrup, Denmark), anti-β-actin-peroxidase 1:20 000 (SIGMA, Cat. No. A3854), anti-STX1 (Santa Cruz, Cat. No. sc-12736), anti-FAM49A (Santa Cruz, Cat. No. sc-390478), anti-GCP5 (TUBGCP5 – Santa Cruz, Cat. No. sc-365837), anti-HP1γ (F-1) (CBX3 – Santa Cruz, Cat. No. sc-398562), anti-Endophilin B2 (A-10) (SH3GLB2 – Santa Cruz, Cat. No. sc-365608), anti-eIF4E (A-10) (Santa Cruz, Cat. No. sc-271480), anti-DISC-1 (B-2) (Santa Cruz, Cat. No. sc-365591). Proteins of interest were visualized with the Amersham^™^ ECL^™^ Detection Reagents (GE Healthcare by SIGMA, Cat. No. RPN2105) or LiteAblot TURBO Extra-Sensitive Chemioluminescent Substrate (EuroClone, Cat. No. EMP012001). Western blotting images were acquired using with Alliance LD2–77WL system (Uvitec, Cambridge) and band intensity was calculated using ImageJ software. FRDA lymphoblasts extracts were prepared in ice-cold lysis buffer (50 mM Tris–HCl pH 7.5, 150 mM NaCl, 1% Igepal CA-630, 5 mM EDTA, 5 mM EGTA) supplemented with Complete Protease Inhibitor Cocktail (Roche Diagnostics). Lysates were clarified by centrifugation, supernatants were mixed with 10× Laemmli sample buffer and boiled for 5 min at 95°C. 50 μg of protein extracts were resolved by 12% SDS-PAGE gels and transferred to 0.2 μM nitrocellulose membrane (Trans-Blot Turbo Transfer pack, Bio-Rad). Membranes were blocked with 5% non-fat dry milk in PBS/0.1% Tween 20 and incubated with the indicated primary and secondary antibodies: mAb anti-FXN (MAB-10876 Immunological Sciences), mAb anti-α-tubulin (clone DM1A, Sigma-Aldrich) and secondary antibody horseradish peroxidase (HRP)-conjugated goat anti-mouse (Thermo Fisher Scientific). The immunoreactive bands were detected by enhanced chemiluminescence (ECL, GE Healthcare) and imaged with a ChemiDoc XRS system (Bio-Rad). Densitometric analysis was performed using ImageLab 5.2 software (Bio-Rad).

### RNA isolation, Reverse Transcription (RT) and quantitative RT-PCR (qRT-PCR)

Total RNA was extracted from cell pellets using TRIzol^®^ Reagent (Thermo Fisher, Cat. No. 15596026) and following manufacturer's instructions. For HEK 293T/17, SH-SY5Y and GM04078 fibroblasts, RNA samples were subjected to TURBO™ DNase (Invitrogen, Cat. No. AM1907) treatment, to avoid plasmid DNA contamination. A total of 1 μg of RNA was subjected to retrotranscription using iScript™cDNA Synthesis Kit (Bio-Rad, Cat. No. 1708890), according to manufacturer's instructions. qRT-PCR was carried out using SYBR green fluorescent dye (iQ SYBR Green Super Mix, Bio-Rad, Cat. No. 1708884) and an iCycler IQ Real time PCR System (Bio-Rad). The reactions were performed on diluted cDNA (1:8). Human GAPDH was used as normalizing control in all qRT-PCR experiments. For GM16214 and GM16215 lymphoblasts, cDNA was prepared using the SuperScript VILO cDNA synthesis kit (Thermo Fisher). Levels of human *FXN* mRNA and miniSINEUPs RNA expression were assessed by real-time qPCR using the StepOne Plus Instrument (Applied Biosystems), normalized with the control genes expression. The assays were performed using the TaqMan primers (Applied Biosystems) listed in Supplementary Table S1. GUSB, GAPDH and ACT were used as control housekeeping genes. The amplified transcripts were quantified using the comparative Ct method and the differences in gene expression were presented as normalized fold expression with ΔΔCt method (44).

### Statistical analysis

In all experiments, the significance of differences between groups was evaluated by one-way ANOVA followed by Dunnett's post-test. Quantitative data are presented as mean ± SEM of at least three independent experiments.

## RESULTS

### Synthetic *SINEUP-FXN*: design and screening

Since SINEUPs target the mRNA sequence around the starting AUG, the precise knowledge of the real sites of transcription initiation (TSS) is crucial, especially in cells and tissues relevant for the gene of interest and its associated disease. In our experience, we have found that the annotation of the reference sequence is often not representative of the cell-type-specific usage of TSSs and of the 5′UTRs of endogenous mRNAs. To build *FXN*-specific SINEUPs, we made use of the FANTOM5 (Functional ANnoTation Of the Mammalian genome) collection of Cap Analysis of Gene Expression (CAGE) datasets, which represents the widest catalogue of annotated promoters and TSSs in mammalian samples (45). Using the Zenbu Genome Browser Tool for data visualization (46), we monitored TSS usage at the human *FXN* locus with a specific focus on human cells lines and tissues relevant for FRDA. Unexpectedly, we found that the annotated reference sequences are poorly representative of human samples. Rather, at least two additional variants (TSS1 and TSS2) of human *FXN* mRNA exist that are positioned more closely to the initiating AUG and are supported by ‘robust’ statistical definition of promoters in FANTOM5. The use of these alternative TSSs finds further confirmation in the Gencode catalogue of transcripts (ENST00000396366, ENST00000498653) (Figure 1A).

Based on TSS analysis, we designed *FXN*-specific SINEUPs (*SINEUP-FXN*) in antisense orientation to the most widely expressed variant of human *FXN* mRNA. By following the pairing rules of the natural BD of AS Uchl1 (37), synthetic BDs were initially designed according to the −40/+32 anatomy (38). Although *FXN* mRNA has a very short 5′ UTRs, we also designed a shorter −40/+4 BD variant, as previously experimentally validated for other targets (40,47). We then generated additional *SINEUP-FXN* by trimming BD sequences at both ends, following a strategy previously used to optimize SINEUPs for ectopically expressed target mRNAs but not yet tested for endogenous genes. Finally, since another methionine (M76) is positioned in-frame in the second exon after the GAA-triplets repeat, we designed three additional BDs overlapping this sequence immediately after or across the exon I/exon II boundary (Figure 1C). Each BD variant was combined with the ED of natural AS Uchl1 that so far represents the most potent translational activator for SINEUPs (Figure 1B).

**Figure 2.**
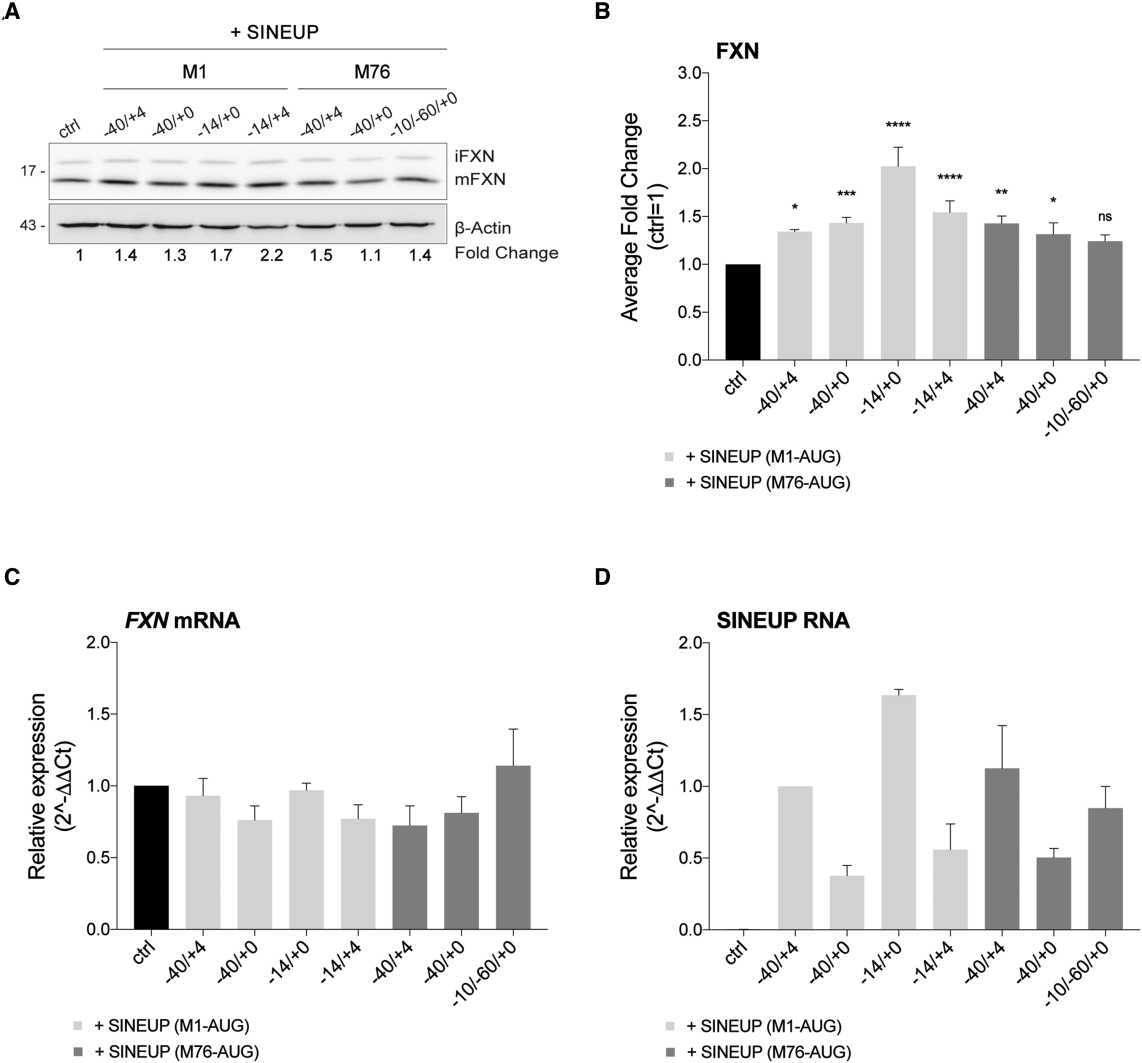
**Synthetic SINEUPs increase quantities of endogenous frataxin *in vitro* in human cells**. (**A-D**) HEK 293T/17 cells were transfected with *SINEUP-FXN* variants and empty vector (ctrl) and harvested 48 hours post-transfection. (**A**) Whole cell lysates were analysed by western blotting with anti-FXN and anti-β-actin antibodies. One representative experiment is shown. First, FXN band intensity was normalized to the relative β-actin band. Then, fold change values were calculated normalizing to control cells (ctrl). *SINEUP-FXN*-transfected cells showed increased levels of endogenous FXN protein. (**B**) Average fold change of FXN protein levels. Columns represent mean ± S.E.M. of *n* ≥ 4 independent experiments; ns, *P* > 0.05; *P < 0.05; **P < 0.01; ***P < 0.001; ****P < 0.0001 (one-way ANOVA followed by Dunnett's post-test). (**C, D**) The real-time RT-PCR analysis of FXN mRNA and SINEUP RNA expression in transfected cells. Columns represent mean ± S.E.M. of *n* ≥ 4 independent experiments. Variation in both target and SINEUP mRNA expression among samples are not statistically significant (one-way ANOVA followed by Dunnett's post-test). (**C**) FXN transcripts were quantified, using human GAPDH (hGAPDH) expression as the internal control. The FXN/hGAPDH ratio for the ctrl sample was set as a baseline value to which all transcripts levels were normalized. Unchanged FXN mRNA levels are shown, thereby confirming FXN increased protein synthesis at the post-transcriptional level. (**D**) SINEUP transcripts were quantified, using the hGAPDH expression as the internal control. The SINEUP/hGAPDH ratio for −40/+4 M1-AUG sample was set as a baseline value to which all transcripts levels were normalized.

To screen the activity of *SINEUP-FXN*, we took advantage of HEK 293T/17 cells which endogenously expresses frataxin starting from the same TSSs of cells relevant for the pathology and have been proven to support SINEUP activity of a variety of SINEUPs targeting endogenous genes (38) [unpublished results]. HEK 293T/17 cells were transfected with *SINEUP-FXN* (+SINEUP) or an empty control vector (ctrl). SINEUP activity was estimated as fold changes in protein levels in +SINEUP compared to control condition by western blotting upon β-actin normalization (Figure 2A). qRT-PCR quantification of *FXN* mRNA was carried out to confirm SINEUPs act at the post-transcriptional levels (Figure 2C). We found that SINEUP activity varies significantly according to the overlapping region. When *SINEUP-FXN* were designed around the initiating M1-AUG as transcribed from both TSS1 and TSS2 and included extra complementary sequences at the target 5′ end, we found that the activity was regulated by the length of the overlap to the coding sequence (CDS). In particular, the configuration of −40/+32 M1-AUG showed no effects on frataxin levels (*data not shown*). Instead, BDs with minimal (−40/+4 M1-AUG) or no (−40/+0 M1-AUG) overlap to the CDS induced up-regulation of mature frataxin in the range of 1.4-fold. When the overlapping region corresponded exactly to the 5′UTR from TSS2, as in the case for −14/+0 and −14/+4 M1-AUG configurations, SINEUPs reached the highest potency (1.5- to 2-fold increase).

When BD was designed at −40/+0 relative to the internal in-frame M76-AUG, we measured an increment lower than 1.5-fold in frataxin protein levels relative to controls cells (ctrl) with high variation among the replicates. This level of up-regulation was also observed for the intron-spanning BD (–10/–60/+0 M76-AUG) although it was not statistically significant.

In summary, we successfully designed synthetic SINEUPs able to increase the quantity of frataxin protein with no effects on its mRNA levels (Figure 2C). SINEUP-mediated up-regulation was in the range of 1.4- to 2.4-fold increase (Figure 2B).

### Synthetic *miniSINEUP-FXN* are active *in vitro*

**Figure 3.**
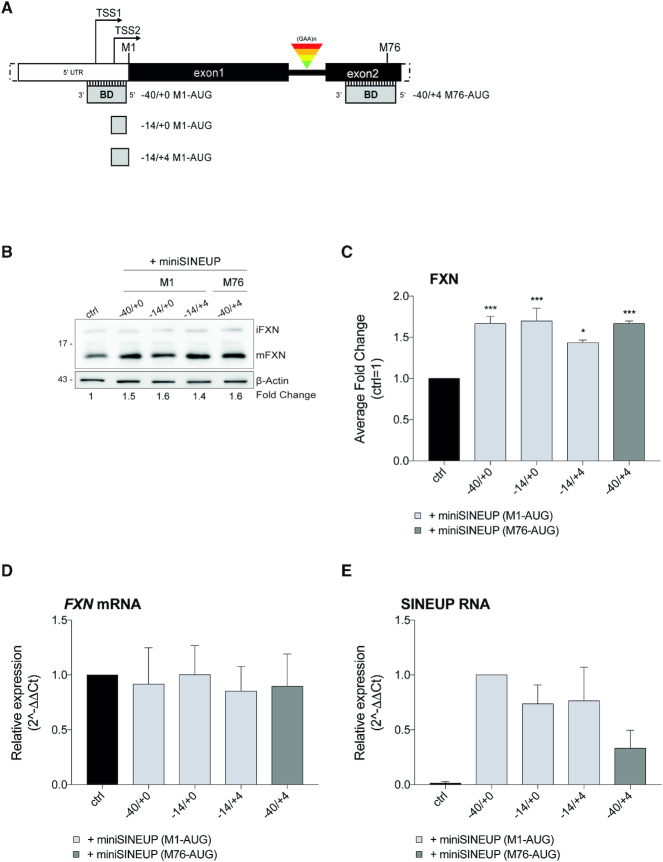
**Synthetic miniSINEUPs increase endogenous FXN protein expression in human cells**. (**A**) Scheme of human *FXN* gene (5′-end) and BDs anatomy of tested synthetic *miniSINEUP-FXN*s targeting the initiating M1-AUG and the M76-AUG downstream GAA expansions. (**B-E**) HEK 293T/17 cells were transfected with *miniSINEUP-FXN* variants (−40/+0; −14/+0 and −14/+4 M1-AUG or −40/+4 M76-AUG) and empty vector (ctrl) and harvested 48 hours post-transfection. (**B**) Whole cell lysates were analysed by western blotting with anti-FXN and anti-β-actin antibodies. One representative experiment is shown. First, FXN band intensity was normalized to the relative β-actin band. Then, fold change values were calculated normalizing to control cells (ctrl). *miniSINEUP-FXN*-transfected cells showed increased levels of endogenous FXN protein. (**C**) Average fold change of FXN protein levels. Columns represent mean ± S.E.M. of *n* = 3 independent experiments; ns, *P* > 0.05; *P < 0.05; **P < 0.01; ***P < 0.001; ****P < 0.0001 (one-way ANOVA followed by Dunnett's post-test). (**D, E**) The real-time RT-PCR analysis of FXN mRNA and miniSINEUP RNA expression in transfected cells. Columns represent mean ± S.E.M. of *n* = 3 independent experiments. Variation in both target and SINEUP mRNA expression among samples are not statistically significant (one-way ANOVA followed by Dunnett's post-test). (**D**) FXN transcripts were quantified, using human GAPDH (hGAPDH) expression as the internal control. The FXN/hGAPDH ratio for the ctrl sample was set as a baseline value to which all transcripts levels were normalized. Unchanged FXN mRNA levels are shown, thereby confirming FXN protein amounts were increased at the post-transcriptional level. (**E**) miniSINEUP transcripts were quantified, using the hGAPDH expression as the internal control. The miniSINEUP/hGAPDH ratio for −40/+0 M1-AUG sample was set as a baseline value to which all transcripts levels were normalized.

Synthetic SINEUPs derived from natural AS Uchl1 RNA are about 1200 nucleotides long. For their potential usage as RNA therapeutics, shorter functional molecules retaining SINEUP translation enhancement activity are needed. Therefore, four different BDs of the most effective *SINEUP-FXN*, −40/+0, −14/+4, 14/+0 on M1-AUG and −40/+4 M76-AUG, were selected and synthetized as miniSINEUPs, producing ≈ 250 nucleotides long transcripts (Figure 3A). They were tested in HEK293T/17 cells as before, proving they maintain the very same activity towards endogenous frataxin (Figure 3B). These results were also confirmed in human neuroblastoma SH-SY5Y cell line (*data not shown*). Altogether, *miniSINEUPs-FXN* promoted a protein induction consistently in the range of 1.4- to 1.7-fold, proving they retain the same efficacy of their full-length counterpart with the advantage of being shorter (Figure 3C).

### Validation of BD specificity and analysis of off-targets

**Figure 4.**
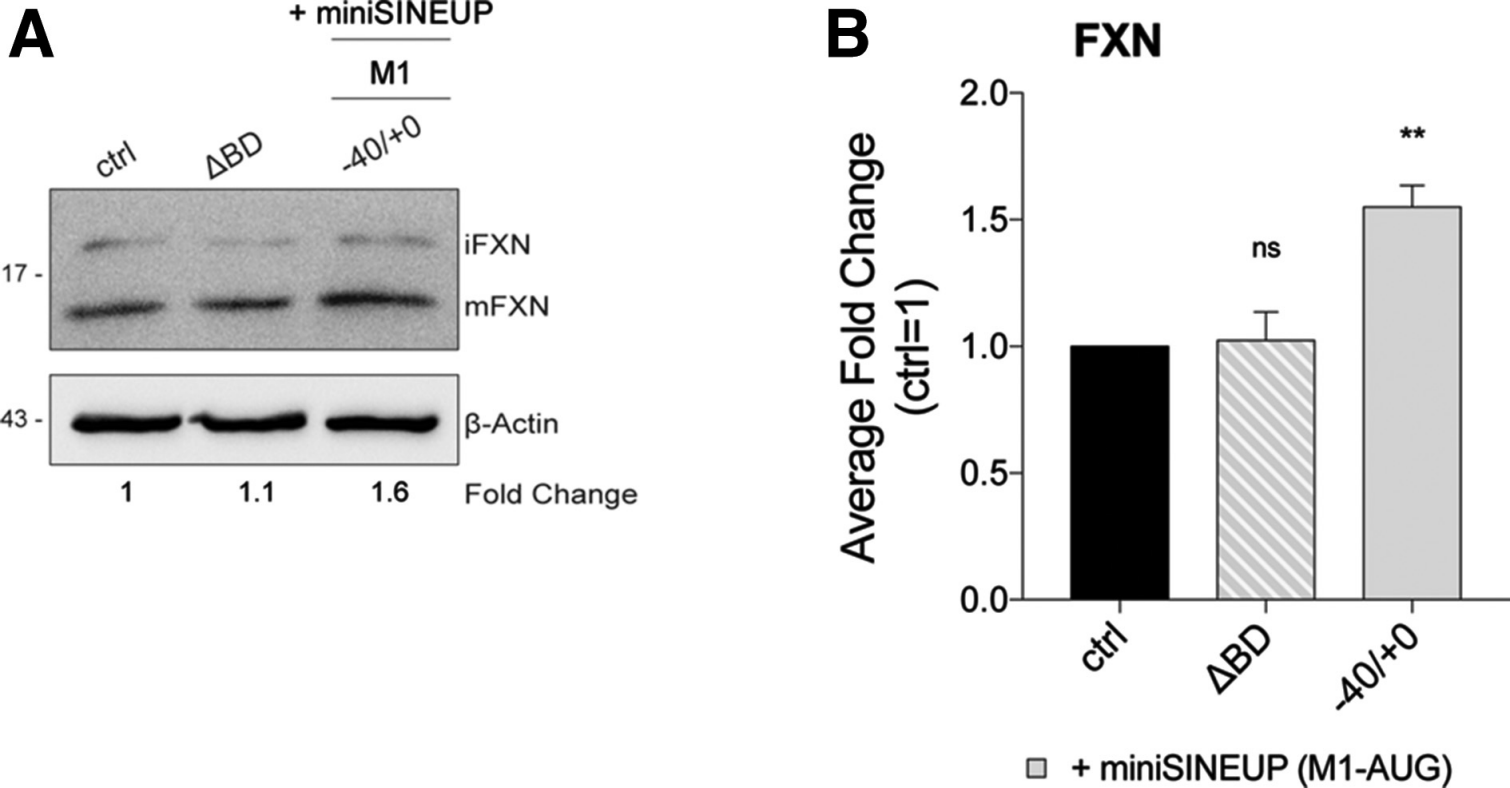
***miniSINEUP-FXN* activity requires the BD sequence**. (**A, B**) HEK 293T/17 cells were transfected with *miniSINEUP-FXN* −40/+0 M1-AUG, ΔBD (deltaBD, construct lacking the overlapping region to *FXN* mRNA) and with empty vector (ctrl) and harvested 48 hours post-transfection. (**A**) Whole cell lysates were analysed by western blotting with anti-FXN and anti-β-actin antibodies. One representative experiment is shown. First, FXN band intensity was normalized to the relative β-actin band. Then, fold change values were calculated normalizing to control cells (ctrl). *miniSINEUP-FXN*-transfected cells showed increased levels of endogenous FXN protein, while ΔBD-transfected cells showed unchanged protein levels. (**B**) Average fold change of FXN protein levels. Columns represent mean ± S.E.M. of *n* ≥ 4 independent experiments; ns, *P* > 0.05; *P < 0.05; **P < 0.01; ***P < 0.001; ****P < 0.0001 (one-way ANOVA followed by Dunnett's post-test).

**Figure 5.**
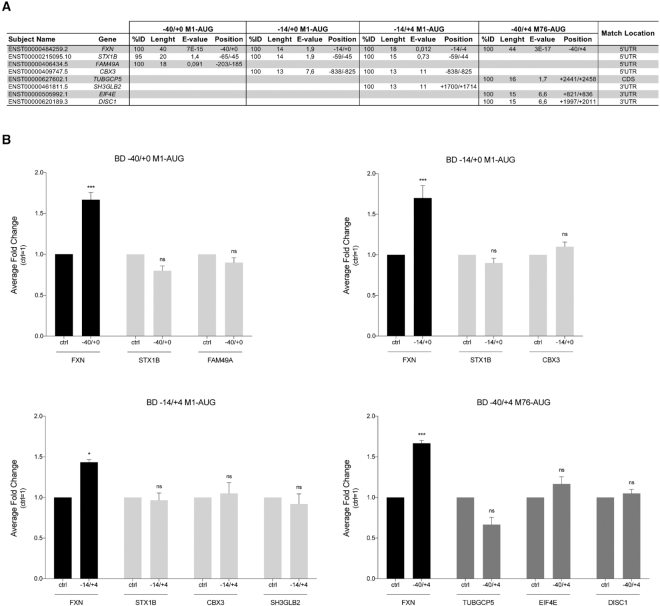
***miniSINEUP-FXN* effects on off-targets protein expression**. (**A, B**) Off-targets analysis. (**A**) Input sequences (BDs) were aligned to the complete transcriptome as available on Ensembl. Selected off-target genes are shown. The selection was based on BDs sequence alignment length and localization (5′, CDS or 3′ relative to the off-target mRNA). (**B**) Off-targets protein expression in transfected cells. HEK 293T/17 cells were transfected with empty vector (ctrl) and miniSINEUP-FXN variants (−40/+0; −14/+0 and −14/+4 M1-AUG or −40/+4 M76-AUG) and harvested 48 hours post transfection. Empty vector (ctrl) was taken as negative control. Whole cell lysates derived from experiments presented in Figure 3 were analysed by western blotting. One representative experiment for each off-target is shown in Supplementary Figure S1. Average fold changes of both target and off-targets for each BD are shown. First, band intensity was normalized to the relative β-actin band. Then, fold change values were calculated normalizing to control cells (ctrl). The mean FXN fold changes are plotted as the mean ± S.E.M. (*n* ≥ 4); ns, *P* > 0.05; *P < 0.05; **P < 0.01; ***P < 0.001; ****P < 0.0001 (one-way ANOVA followed by Dunnett's post-test). Off-targets fold changes are plotted as the mean ± SEM (*n* = 3) and p values are calculated by unpaired t-test with Welch's correction. ns, *P* > 0.05. Unchanged off-targets protein levels are shown.

SINEUPs and miniSINEUPs specificity for the target is guaranteed by the antisense sequence complementarity between the mRNA and BDs. To demonstrate the dependency of *miniSINEUP-FXNs* activity on sequence pairing, we synthesized a miniSINEUP lacking the BD (deltaBD, ΔBD). As expected, no effects on FNX protein levels were detected (Figure 4). However, the possibility of off-targets pairing, and the subsequent up-regulation of unintended proteins cannot be ruled out. To predict putative off-targets, we took advantage of the Basic Local Alignment Search Tool (BLAST) of Ensembl genome browser to align the BD sequences to human mRNA dataset. Results were filtered for match orientation while non-functional genes (pseudogenes and patch chromosomes) were removed. As listed in Figure 5A, potential off-targets mRNAs were identified for their 100% identity to *SINEUP-FNXs* within the 5′UTRs of *STX1B, FAM49A* and *CBX3* genes with length ranging from 13 to 20 nts. All complementary sequences were distant from the translation initiation site of the target mRNA. Unconventional positions of target binding sites were also identified for *TUBGCP5* (CDS) and for *SH3GLB2, EIF4E* and *DISC1* (3′UTRs). As shown in Figure 5B and Supplementary Figure S1, in the very same lysates where *miniSINEUP-FNXs* were active, no effects on these additional targets were observed.

### Synthetic *miniSINEUP-FXNs* rescue physiological levels of frataxin protein in FRDA fibroblasts

**Figure 6.**
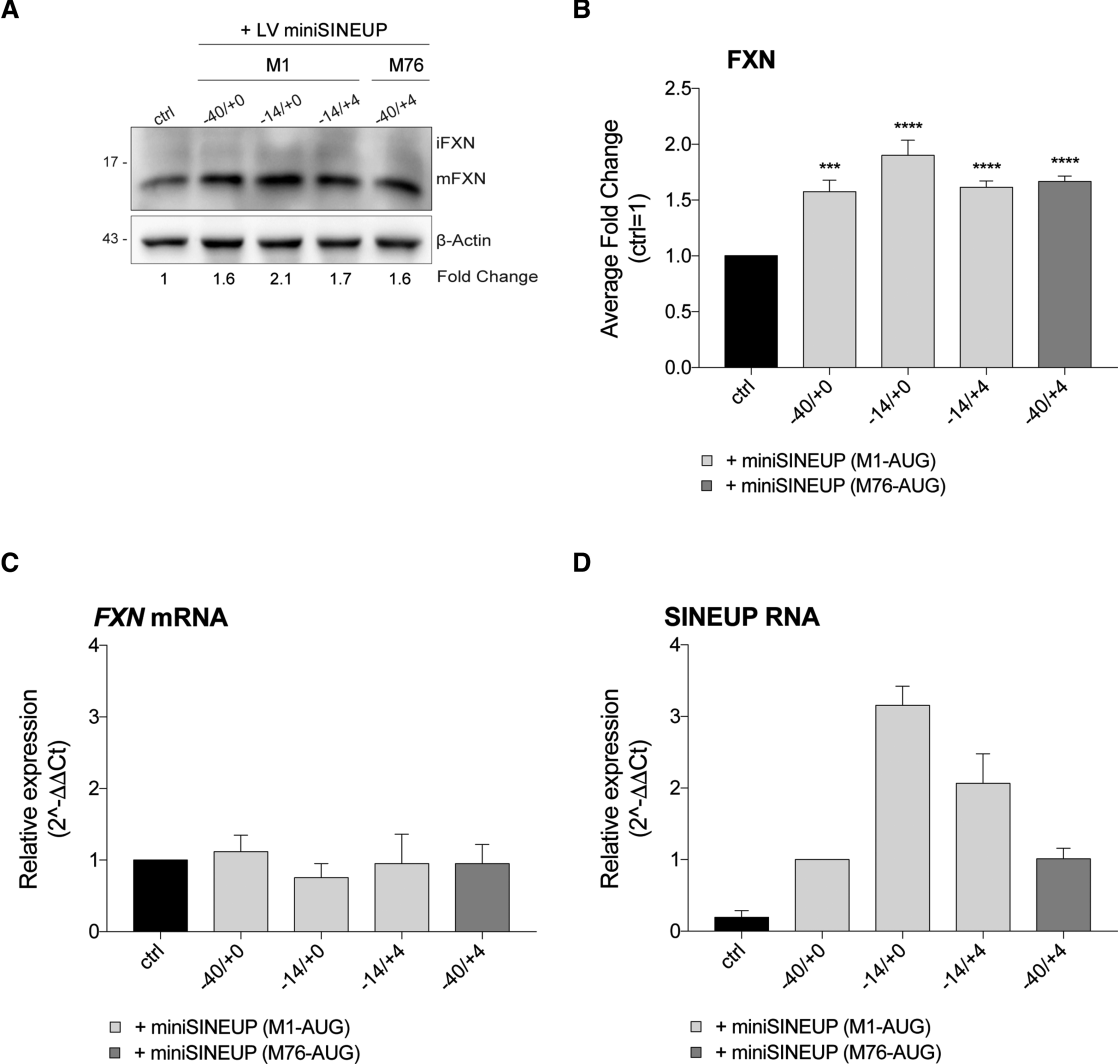
**Rescue of frataxin protein levels in FRDA patient-derived fibroblasts**. (**A-D**) GM04078 cells (patients’ primary fibroblasts) were infected with inducible lentiviral vectors driving the expression of *LVminiSINEUP-FXN* variants (−40/+0; −14/+0 and −14/+4 M1-AUG or −40/+4 M76-AUG) and empty viral particles (ctrl), induced 48 and 96 hours post-infection and harvested 6 days post-infection. (**A**) Whole cell lysates were analysed by western blotting with anti-FXN and anti-β-actin antibodies. One representative experiment is shown. First, FXN band intensity was normalized to the relative β-actin. Then, fold change values were calculated normalizing to control cells (ctrl). *LVminiSINEUP-FXN*-infected cells show increased levels of endogenous FXN protein. (**B**) Average fold change of FXN protein levels. Columns represent mean ± S.E.M. of *n* ≥ 4 independent experiments; ns, *P* > 0.05; *P < 0.05; **P < 0.01; ***P < 0.001; ****P < 0.0001 (one-way ANOVA followed by Dunnett's post-test). (**C, D**) The real-time RT-PCR analysis of FXN mRNA and miniSINEUP RNA expression in transfected cells. Columns represent mean ± S.E.M. of n≥4 independent experiments. Variation in both target and miniSINEUP mRNA expression among samples are not statistically significant (one-way ANOVA followed by Dunnett's post-test). (**C**) FXN transcripts were quantified, using human GAPDH (hGAPDH) expression as the internal control. The FXN/hGAPDH ratio for the ctrl sample was set as a baseline value to which all transcripts levels were normalized. Unchanged FXN mRNA levels are shown. (**D**) miniSINEUP transcripts were quantified, using the hGAPDH expression as the internal control. The miniSINEUP/hGAPDH ratio for −40/+0 M1-AUG sample was set as a baseline value to which all transcripts levels were normalized.

FRDA-derived cells represent the most relevant cellular model to test therapeutic strategies for patients, as they carry the complete *FXN* locus together with pathogenic GAA-triplet repeat expansions (48). All *miniSINEUP-FXN* constructs were cloned into a lentiviral expression vector (LV), in-between the doxycycline-controlled TREt promoter and BGHpA to build inducible recombinant lentiviral particles (LVs *miniSINEUP-FXN*). We took advantage of LVs, paired with a constitutive rtTA2S-M2 trans-activator, to drive delayed and TetON-controlled expression of *miniSINEUP-FXN*. An additional virus, lacking miniSINEUP, was used as a negative control (ctrl). To optimize induction conditions, lentiviral particles were transduced in SH-SY5Y cells, and miniSINEUP expression and frataxin levels were measured at different time points, following a single or a double doxycycline induction (Supplementary Figure S2A). We found that a single doxycycline stimulation, combined with tests at 48 h, was not sufficient to trigger SINEUP-mediated increase in frataxin quantities. Instead, a double treatment protocol was crucial to promoting protein induction (Supplementary Figure S2B, C). We then applied the double doxycycline stimulation protocol to infected FRDA cells as a proof-of-principle that SINEUPs could be exploited in a pathological context. Among available patients-derived cells, we took advantage of primary FRDA fibroblasts (GM04078) showing an intermediate phenotype carrying a hyper-expansion of about 541 repeats on one allele and 420 repeats on the other one. All LVs miniSINEUPs led to an increase in frataxin quantities in the range of 1.6- to 2.1 fold (Figure 6). Importantly, the position of SINEUP BD relative to the GAA expansion and the presence of the pathological expansion itself did not interfere with the observed protein increase in patients’ cells. Considering that GM04078 cells show reduced levels of frataxin, averaging around 40% when compared to age- or sex-matched healthy-derived cells (49), SINEUP activity rescued physiological protein quantities in this FRDA cellular model.

### Synthetic *miniSINEUP-FXN* rescue physiological levels of frataxin protein in FRDA lymphoblasts

The ability of *miniSINEUP-FXN* to up-regulate endogenous frataxin levels in patient's fibroblasts prompted us to evaluate their therapeutic potential in a different FRDA cellular model. To this purpose, we employed FRDA-derived lymphoblasts, which carry the pathogenic expansion of GAA repeats and show reduced levels of the protein when compared to controls.

**Figure 7.**
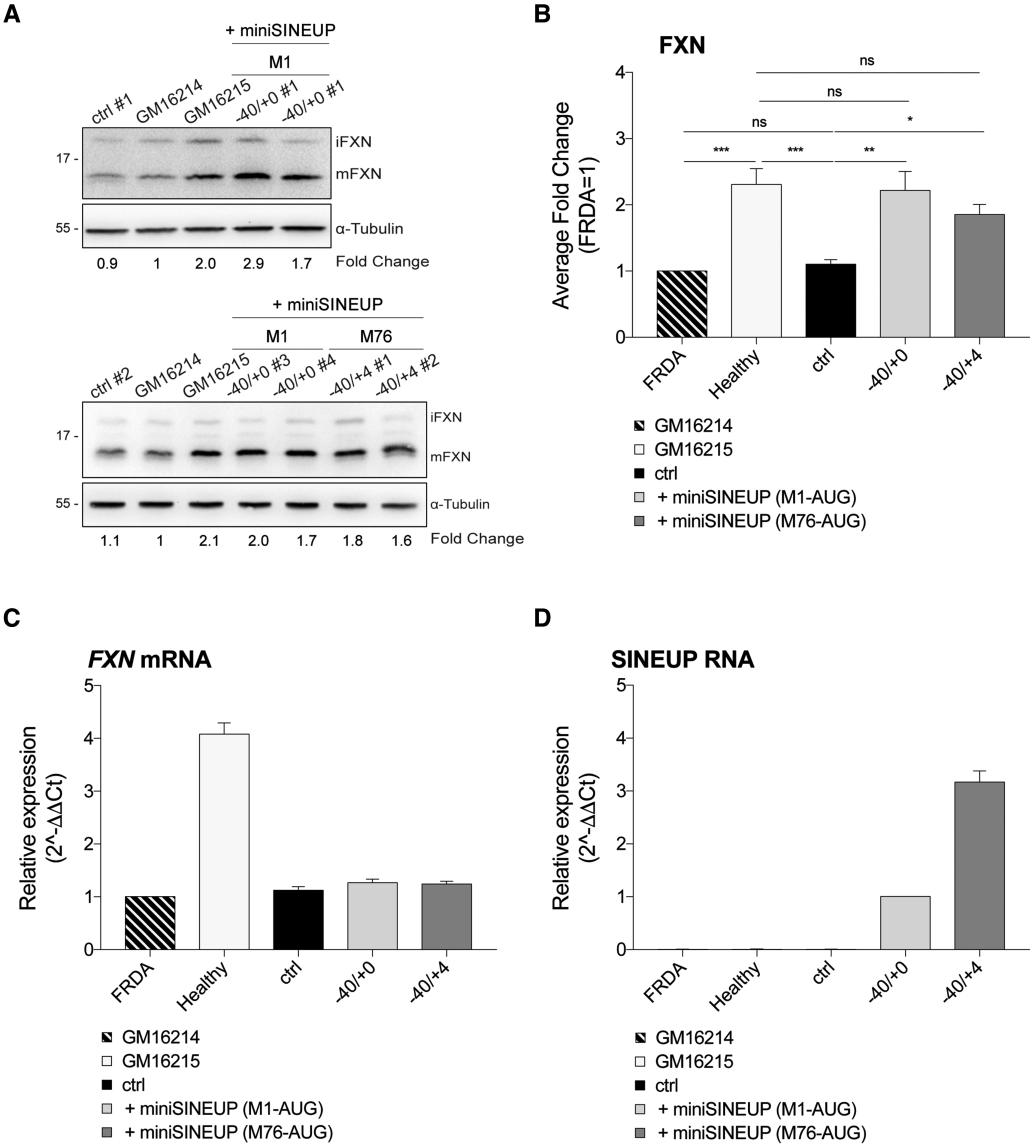
**Rescue of frataxin protein levels in FRDA patient-derived lymphoblasts**. (**A-D**) GM16214 cells (patients’ primary lymphoblasts) were stably transfected with *miniSINEUP-FXN* variants and empty vector (ctrl). *miniSINEUP-FXN* (−40/+0 M1-AUG), *miniSINEUP-FXN* (−40/+4 M76-AUG) and ctrl stable clones were obtained from at least 15 days of G418 selection. (**A**) Whole cell lysates were analysed by western blotting with anti-FXN and anti-β-actin antibodies. Two representative experiments are shown. First, FXN band intensity was normalized to the relative β-actin. Then, fold change values were calculated normalizing to GM16214 cells. GM16214 cells expressing *miniSINEUP-FXN* show increased levels of endogenous FXN protein. (**B**) Average fold change of FXN protein levels. Columns represent mean ± S.E.M. of *n* ≥ 4 independent experiments; ns, *P* > 0.05; *P < 0.05; **P < 0.01; ***P < 0.001; ****P < 0.0001 (one-way ANOVA followed by Dunnett's post-test). (**C, D**) The real-time RT-PCR analysis of FXN mRNA and miniSINEUP RNA expression in transfected cells. Columns represent mean ± S.E.M. of n≥4 independent experiments. Variation in both target and miniSINEUP mRNA expression among samples are not statistically significant (one-way ANOVA followed by Dunnett's post-test). (**C**) FXN transcripts were quantified, using human GAPDH (hGAPDH) expression as the internal control. The FXN/hGAPDH ratio for the GM16214 sample was set as a baseline value to which all transcripts levels were normalized. Unchanged FXN mRNA levels are shown. (**D**) miniSINEUP transcripts were quantified, using the hGAPDH expression as the internal control. The miniSINEUP/hGAPDH ratio for −40/+0 M1-AUG sample was set as a baseline value to which all transcripts levels were normalized.

Frataxin-deficient lymphoblasts derived from an FRDA patient (GM16214) were stably transfected either with the *miniSINEUP-FXN* targeting the initiating AUG (−40/+0 M1-AUG) or an empty vector as the negative control (ctrl). Following antibiotic selection, independent clonal subpopulations were monitored by western blot and real-time RT-PCR analysis (Figure 7A, C-D). Frataxin protein levels were evaluated in whole cell extracts from different clones and compared to untransfected lymphoblasts. As expected, extracts from FRDA cells showed a significant deficit of FXN protein expression averaging ∼2.3-fold when compared to control lymphoblasts derived from the healthy heterozygous patient's mother (Figure 7B). Analysis of independent miniSINEUP clones revealed a strong rescue of frataxin levels while negative control transfectants (ctrl) showed no significative change. In particular, we observed an up-regulation ranging from 1.6- to 2.9-fold when compared to negative controls (Figure 7B). *FXN* mRNA expression was quite similar in FRDA patient cells, negative control clones and *miniSINEUP-FXN* clones, confirming SINEUPs’ post-transcriptional mode of action (Figure 7C). Next, we investigated whether a *miniSINEUP-FXN* targeting the AUG downstream GAA expansions (−40/+4 M76-AUG) could exert the same activity in these cells. To this purpose, we generated new stably transfected FRDA cells. As shown in Figure 7A, B, this *miniSINEUP-FXN* was able to increase frataxin protein levels from 1.6- to 2.0-fold. According to their activity at the step of translation, *FXN* mRNA levels never showed significant changes (Figure 7C).

### Functional rescue of a disease-associated phenotype in FRDA patient cells

**Figure 8.**
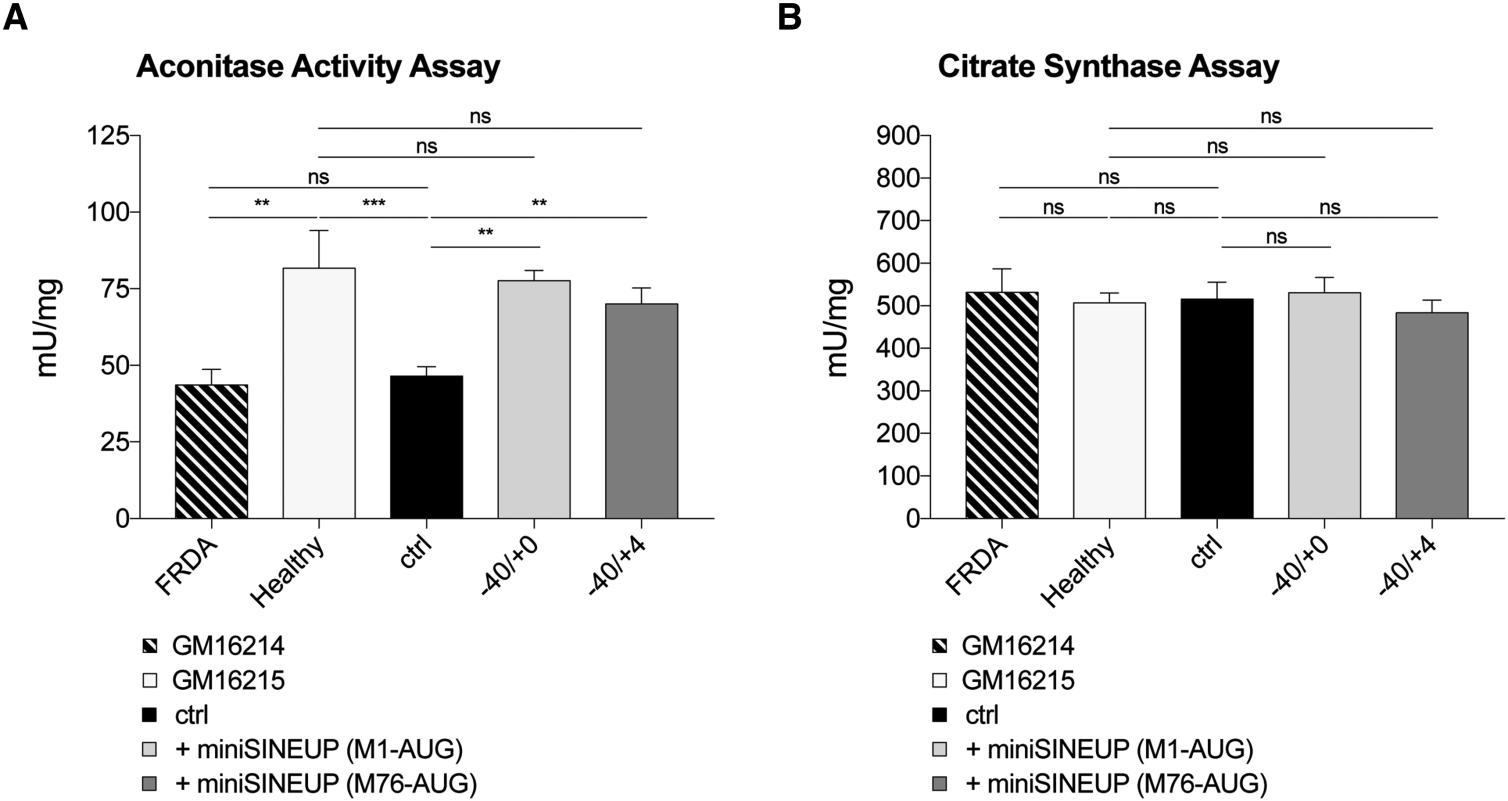
**Rescue of aconitase defects in FRDA patient-derived lymphoblasts**. (**A, B**) The effect of *miniSINEUP-FXN* expression on aconitase activity was measured on whole cells lysates. GM16214 cells expressing *miniSINEUP-FXN* showed restored activity of endogenous aconitase as compared to GM16215 positive control. Aconitase (**A**) and citrate synthase (**B**) activities are expressed as mU/mg ratio. Citrate synthase assay is used as an internal control. Columns represent mean ± S.E.M. (*n* ≥ 4) of mU/mg values; ns, *P* > 0.05; *P < 0.05; **P < 0.01; ***P < 0.001; ****P < 0.0001 (one-way ANOVA followed by Dunnett's post-test).

As previously reported by several studies, frataxin-deficient cells are primarily affected by defective ISC biosynthesis. Accordingly, insufficient frataxin levels trigger a typical loss in the activity of aconitases, two different ISC-dependent enzymes located in mitochondrial and cytosolic compartments. To assess the functional impact of SINEUPs, aconitase activity was chosen as a functional readout of restoring frataxin physiological levels on the above-described FRDA stable transfectants. To evaluate the status of aconitases, enzyme activity was monitored in total lysates from *miniSINEUP-FXN* clones and untransfected lymphoblasts by spectrophotometric assays. Extracts from these FRDA-derived cells exhibited a loss of aconitase activity close to 50% when compared to healthy-derived cells from the patient's parent. Total aconitase activity was rescued in FRDA lymphoblasts stably expressing *miniSINEUP-FXN* targeting the initiating AUG (−40/+0 M1-AUG) while it remained at pathological levels in control cells. Furthermore, the deficit was also rescued in cells expressing the *miniSINEUP-FXN* variant targeting the internal AUG (−40/+4 M76-AUG) (Figure 8A). Measured as a control, activity of citrate synthase, the Krebs cycle enzyme catalyzing the preceding step respect to aconitase, but lacking ISC, did not show significant fluctuations in assayed extracts (Figure 8B). These results demonstrate the rescue of the major disease-associated phenotype in cells derived from an FRDA patient. Altogether, our data prove that treatment with miniSINEUPs targeting frataxin achieved protein physiological levels and a consistent rescue of pathophysiological defects in FRDA-derived cells.

## DISCUSSION

FRDA is a rare genetic disorder caused by an insufficient quantity of frataxin protein. Since its discovery in 1996, the understanding of frataxin functions has grown rapidly (16). However, no effective therapy is currently available to patients (50). The main root of the pathology is the impaired transcription of the *FXN* gene as a result of GAA repeat expansion. Disease onset, severity, and rate of progression are strictly dependent on the length of repeat expansion and ultimately frataxin levels. Homozygous GAA expansion leads to a pronounced drop in protein levels, up to only 20–30% compared to noncarriers healthy individuals (51,52).

During the last decade, RNA-based therapy had a burst of interest for both the high selectivity and the potential scalability to treat a large repertory of human diseases. Most RNA therapeutic molecules are inhibitory RNAs and have been developed to down-regulate the expression of pathogenic genes. Among them, some Small Inhibitory RNAs (siRNAs) and Anti-Sense Oligonucleotides (ASOs) are already approved for clinical use (53). On the other hand, a large group of diseases would strongly benefit from transcriptional- or translation-stimulating drugs able to rescue physiological levels of a specific target protein (54). Recently, gene-specific transcriptional activating RNAs (RNAa) (55) and non-degradative ASOs (56) have been employed to increase the expression of selected genes. In the case of frataxin, almost all investigated molecules are currently aiming at increasing *FXN* transcription (57–59). By acting at post-transcriptional levels, SINEUP technology represents a new and alternative approach to increase *FXN* expression. In addition, SINEUPs present several advantages: target gene expression is induced within the range of 1.5- to 2.5-fold thus limiting side effects due to exaggerated overexpression; SINEUP enables exclusively *in situ* translation enhancement avoiding ectopic protein synthesis in the absence of the target mRNA. Importantly, SINEUPs do not trigger any hereditable genome editing.

Given their modular structure, different synthetic *SINEUP-FXN* have been designed to specifically increase *FXN* translation with BDs in antisense orientation 5′ head-to-head to the most widely expressed *FXN* mRNA. Starting from the anatomy of the BD of AS Uchl1, the representative transcript of natural SINEUPs (−40/+32 M1-AUG), we screened different BDs to identify the ones that were shorter while maintaining specificity. Each variant was combined with the ED of AS Uchl1 that so far represents the most potent translational activator for SINEUPs.

In summary, we show that synthetic SINEUPs against the endogenous mRNA of *FXN* are able to increase its protein synthesis. Furthermore, we demonstrate BD’s flexibility in designing them around the initiating AUG of target mRNA as well as covering an internal in-frame methionine (M76-AUG). While AUG overlap is crucial to retain SINEUP activity when targeting over-expressed mRNAs (40), we prove it is not required in the case of endogenous transcripts. In addition, a BD as short as 14 nts, still confers strong SINEUP activity. This is strictly dependent on sequence homology with target mRNA since a SINEUP molecule lacking BD is not active. Interestingly, proteins levels of potential off-targets sharing homology with the BDs of *SINEUP-FXN* remained unchanged. In the case of *TUBGCP5, SH3GLB2, EIF4E* and *DISC1* this is not surprising since target sequences are located in the non-conventional CDS and 3′UTRs regions. On the other hand, despite target sequences are in their 5′UTRs, no induction was observed for *STX1B, FAM49A* and *CBX3* proteins. Interestingly, the homology regions are all located far from the AUG suggesting that this feature may be crucial for a correct design of an active synthetic SINEUP. Further data on SINEUPs for a large number of targets will better define the requirements for the localization of the homology region in mRNAs.

Together with the demonstration that SINEUPs and miniSINEUPs are the first molecules to positively regulate frataxin at post-transcriptional level, SINEUP activity in FRDA-derived fibroblasts and lymphoblasts was shown to be sufficient to re-establish frataxin physiological levels with an increase in the range of 2-fold of the amounts found in untreated cells.

Frataxin is an activator of the ISC biosynthetic machinery (18–20) and an iron-chaperone (60–62). Furthermore, frataxin can physically interact with mitochondrial and cytosolic aconitase, with the ability to protect or reactivate the enzyme's cofactor (62,63). Accordingly, the rescue of aconitase defects has been associated with a therapeutic increase of frataxin levels in cellular and animal models of FRDA (30,64–66). It was therefore important to prove that the increase of frataxin protein mediated by miniSINEUPs was able to rescue the activity of ISC-dependent enzymes to physiological levels in patient's lymphoblasts.

A great effort is undergoing on the optimization of advanced carrier nanoparticles and/or new-generations of adenoviral-associated vectors (AAV) to meet the specific challenges of reaching the central nervous system (CNS). Furthermore, chemically-modified nucleic acid-based drugs can be directly delivered into the brain by intrathecal injection. Thus, among others, a modified ASO targeting ISS-N1 (Intronic Splicing Silencer N1) has been successfully administrated to the human CNS by intrathecal injection (67) and it has been recently approved by Food and Drug Administration (FDA) for the treatment of spinal muscular atrophy.

Two different strategies can be envisioned for the delivery of SINEUPs *in vivo*. By taking advantage of an appropriate serotype of AAV and a strong promoter, SINEUP RNA can be expressed in the brain upon stereotaxic AAV injection. On the other hand, chemically-modified SINEUPs could be delivered intrathecally. In this case, a crucial limiting factor concerns an excessive length of the molecule so that cells’ entry is inefficient (68). To confront these requirements, *miniSINEUP-FXNs* were shown to be as active as the longer *SINEUP-FXN*s shortening SINEUP RNA to less than 250 nts (38). Chemical footprinting of invSINEB2 has recently identified several structural regions that are required for the ability of AS Uchl1 to increase translation, including a short terminal stem-loop hairpin structure (SL1) as key structural determinant (69). Therefore, it is tempting to speculate that shorter active EDs could be designed based on structural data. The combination of 14 nts long BD with fragmented EDs may lead to active antisense SINEUPs (ASO-SINEUP) short enough for chemical synthesis and delivery *in vivo*.

In conclusion, we provide strong evidence that synthetic SINEUPs enhance endogenous protein expression to the physiological range in a cellular model of a monogenic disease. Their ability to revert pathophysiological phenotypes in human patients’ cells warrants the pre-clinical evaluation of a SINEUP-based therapy to treat FRDA. More broadly, these evidences support that synthetic SINEUPs represent a scalable platform to treat haploinsufficiency disorders.

## Supplementary Material

gkz798_Supplemental_File
