# Supplementary material for: SINEUP non-coding RNAs rescue defective frataxin expression and activity in a cellular model of Friedreich's Ataxia
Source: Nucleic Acids Res. 2019 Oct 4;47(20):10728–43. doi: 10.1093/nar/gkz798 (PMC6847766; doi:10.1093/nar/gkz798)
Supplement: gkz798_Supplemental_File [file gkz798_supplemental_file.pdf]

Supplementary Table 1

Cloning Oligo:	Sequence (5’ → 3’):	Features:
-40/+4 fwd	*TCGAGGCTGCTCCGGGTCTGCCGCCCGCTCCGCCCTCCAGCGCTG*	BD of SINEUP and miniSINEUP
-40/+4 rev	*CAGCGCTGGAGGGCGGAGCGGGCGGCAGACCCGGAGCAGCC*	BD of SINEUP and miniSINEUP
-40/+0 fwd	*TCGAGGCTGCTCCGGGTCTGCCGCCCGCTCCGCCCTCCAGCGCTG*	BD of SINEUP and miniSINEUP
-40/+0 rev	*CAGCGCTGGAGGGCGGAGCGGGCGGCAGACCCGGAGCAGCC*	BD of SINEUP and miniSINEUP
-14/+0 fwd	*TCGAGGCTGCTCCGGGTCT*	BD of SINEUP and miniSINEUP
-14/+0 rev	*AGACCCGGAGCAGCC*	BD of SINEUP and miniSINEUP
-14/+4 fwd	*TCGAGACATGCTGCTCCGGGTCT*	BD of SINEUP and miniSINEUP
-14/+4 rev	*AGACCCGGAGCAGCATGTC*	BD of SINEUP and miniSINEUP
-40/+4 fwd	*TCGAGTCATCAAATAGACACTCTGCTTTTTGACATTCCAAATCTGGTTG*	BD of SINEUP and miniSINEUP
-40/+4 rev	*CAACCAGATTTGGAATGTCAAAAAGCAGAGTGTCTATTTGATGAC*	BD of SINEUP and miniSINEUP
-40/+0 fwd	*TCGAGCAAATAGACACTCTGCTTTTTGACATTCCAAATCTGGTTG*	BD of SINEUP and miniSINEUP
-40/+0 rev	*CAACCAGATTTGGAATGTCAAAAAGCAGAGTGTCTATTTGC*	BD of SINEUP and miniSINEUP
-10/-60/+0 fwd	*TCGAGCAAATAGACACTCTGCTTTTTGACATTCCAAATCTGGTTGAGGCCACGTTGGTTCGAACTTGCGCGGCG*	BD of SINEUP and miniSINEUP
-10/-60/+0 rev	*CCGCCGCGCAAGTTCGAACCAACGTGGCCTCAACCAGATTTGGAATGTCAAAAAGCAGAGTGTCTATTTGC*	BD of SINEUP and miniSINEUP

Syber Green Oligo:	Sequence (5’ → 3’):	Features:
hGAPDH fwd	*TCTCTGCTCCTCCTGTTC*	Housekeeping gene
hGAPDH rev	*GCCCAATACGACCAAATCC*	Housekeeping gene
mAS3' Uchl1 fwd	*CTGGTGTGTATTATCTCTTATGC*	ED (SINEUP)
mAS3' Uchl1 rev	*CTCCCGAGTCTCTGTAGC*	ED (SINEUP)
pTSinvB2 fwd	*CAGTGCTAGAGGAGGTCAGAAGA*	ED (miniSINEUP)
pTSinvB2 rev	*GGAGCTAAAGAGATGGCTCAGCACTT*	ED (miniSINEUP)
hFXN fwd1	*GTGATCAACAAGCAGACGCCAAACAAGCA*	FRDA Fibroblast
hFXN rev1	*GTACACCCAGTTTTTCCCAGTCCAGTCA*	FRDA Fibroblast
hFXN fwd2	*CCTTGCAGACAAGCCATACACGTTTGAG*	FRDA Fibroblast
hFXN rev2	*CTGCTTGTTGATCACATAGGTTCCTAGATC*	FRDA Fibroblast
hFXN fwd	*GGAAACGCTGGACTCTTTAGC*	Human Cell lines
hFXN rev	*CCAGTTTGACAGTTAAGACACCA*	Human Cell lines
hStx1b fwd	*TTGGGAGACATCAGAGCAGG*	off-target
hStx1b rev	*GACGCATAGAAAGGGCACAG*	off-target
hFam49a fwd	*CGTCCAAGACTTACGGGTGG*	off-target
hFam49a rev	*CCTTGGGTCTGTAATGGCCT*	off-target
hTubgcp5 fwd	*GCTCAGTTCGGACCACAAAA*	off-target
hTubgcp5 rev	*TGTAGTTGTGCAAGCTGTTCA*	off-target

TaqMan Oligo:	Sequence (5’ → 3’):	Features:
FXN	Hs00175940_m1	Target
hGAPDH	Hs99999905_m1	Housekeeping gene
GUSB	Hs00939627_m1	Housekeeping gene
ACTB	Hs99999903_m1	Housekeeping gene
miniSINEUP ED fwd	GGTCAGAAGAGGGCATTGGA,	ED (miniSINEUP)
miniSINEUP ED rev	CCACCACGAGGTTACCGTATAAC	ED (miniSINEUP)
Probe	CCCCCAGAACTGG	ED Probe

Supplementary Figure 1

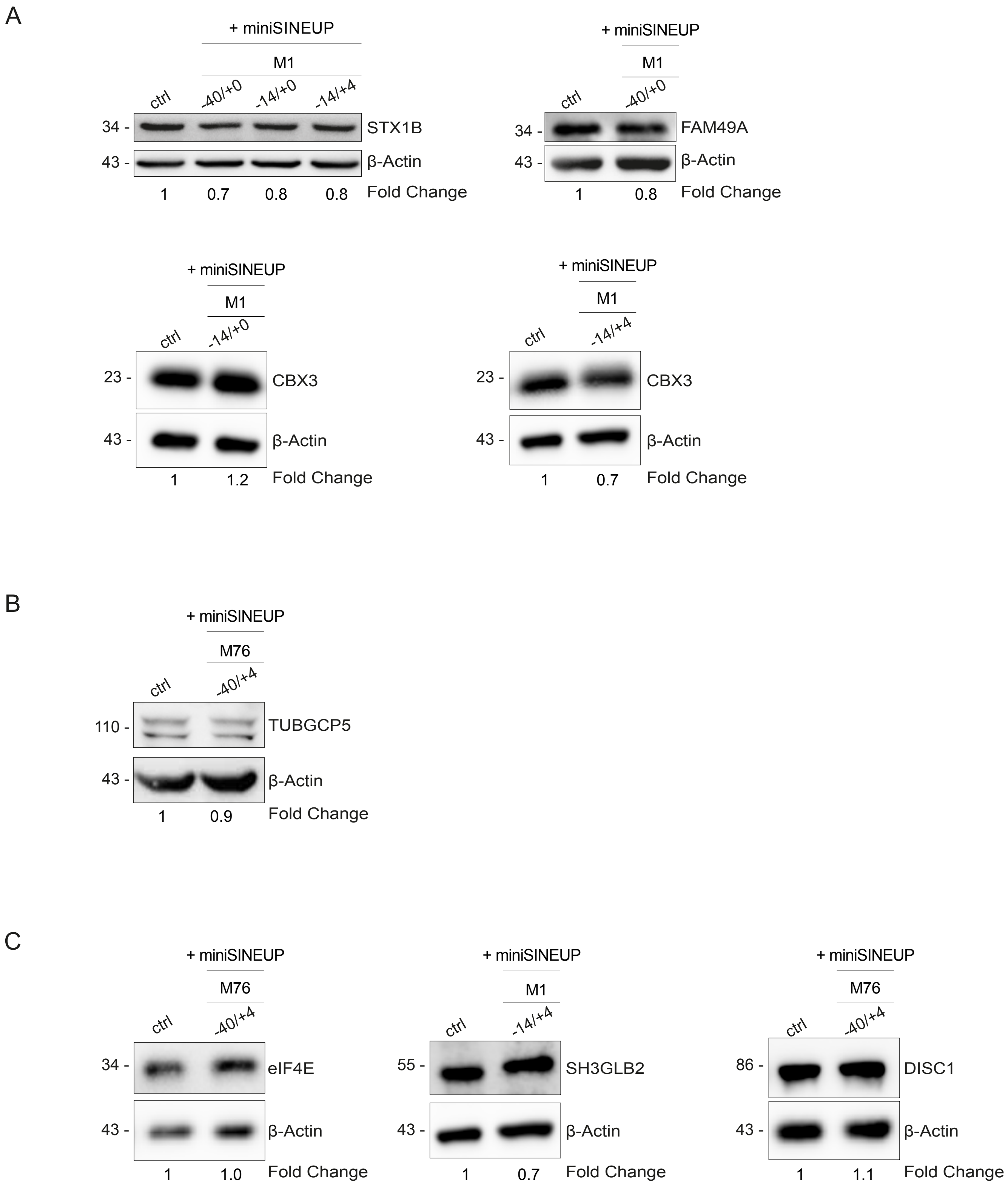

Supplementary Figure 2

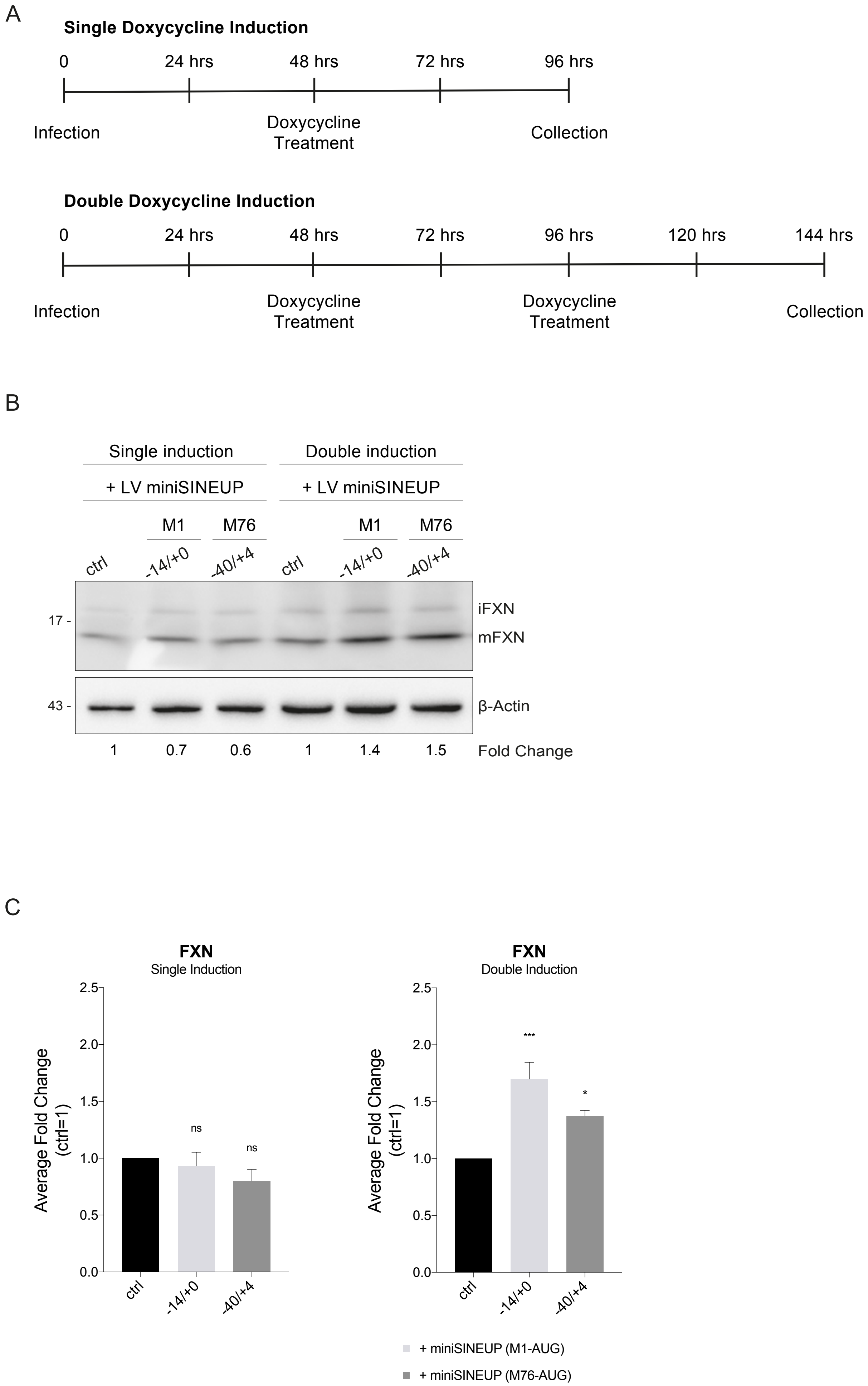

Supplementary material

Supplementary Table 1. List of primers. Complete list of oligonucleotides used in this study for cloning and quantitative PCR experiments.

Supplementary Figure 1. **Off-targets western blot analysis.** (**A-C**) HEK 293T/17 cells were transfected with empty vector (ctrl) and miniSINEUP-FXN variants (-40/+0; -14/+0 and -14/+4 M1-AUG or -40/+4 M76-AUG) and harvested 48 hours post transfection. Empty vector (ctrl) was taken as negative control. Whole cell lysates derived from experiments presented in **Figure 3** were analysed by western blotting with anti-STX1B, anti-FAM49A, anti-CBX3 in (**A**), anti-TUBGCP5 in (**B**), anti-EIF4E, anti-SH3GLB2, anti-DISC1 in (**C**) and anti-β-actin antibodies. Off-targets western blot are grouped on the basis of alignment localization: 5'-UTR in (**A**), CDS in (**B**) and 3'-UTR in (**C**). One representative experiment for each off-target is shown. First, band intensity was normalized to the relative β-actin band. Then, fold change values were calculated normalizing to control cells (ctrl). Average fold changes are shown in **Figure 5B**. Unchanged off-targets protein levels are shown.

Supplementary Figure 2. **miniSINEUPs lentiviral transduction optimization. (A-C)** Infection of human neuroblastoma cells (SH-SY5Y) with inducible lentiviral vectors driving the expression of LVminiSINEUP-FXN variants (-14/+0 M1-AUG or -40/+4 M76-AUG) and empty viruses (ctrl) **(A)** Doxycycline treatment timelines. Single induction (top): 48 hrs after infection (time 0), cells were subjected to doxycycline treatment and harvested 96 hrs after infection. Double induction (bottom): cells were treated twice with doxycycline (time 48 and 96 hrs) and harvested 144 hrs after infection. **(B)** SH-SY5Y cells were infected following both protocols timing. Whole cell lysates were analysed by western blotting with anti-FXN and anti-β-actin antibodies. One representative experiment is shown. First, FXN band intensity was normalized to the relative β-actin. Then, fold change values were calculated normalizing to control cells (ctrl). LVminiSINEUP-FXN-infected cells show increased levels of endogenous FXN protein only with double doxycycline induction, while no up-regulation was observed after a single treatment. **(C)** Average fold change of FXN protein levels. Columns represent mean ± S.E.M. of $n \geq 3$ independent experiments; ns, $p>0.05$; *$p<0.05$; **$p<0.01$; ***$p<0.001$; ****$p<0.0001$ (one-way ANOVA followed by Dunnett's post-test).
